# Challenges in Management of Kidney Failure in a Free Clinic Setting: A Case Report

**DOI:** 10.7759/cureus.26352

**Published:** 2022-06-26

**Authors:** Nicholas Blackmond, Tiffany Loh, Joshua D Kanke, Urmilla Khilanani, Raymond Weitzman

**Affiliations:** 1 Internal Medicine, Dr. Gary Burnstein Community Health Clinic, Pontiac, USA; 2 Internal Medicine, Oakland University William Beaumont School of Medicine, Rochester Hills, USA; 3 Pharmacy, College Pharmacy, Colorado Springs, USA; 4 Rheumatology, Dr. Gary Burnstein Community Health Clinic, Pontiac, USA

**Keywords:** diabetes and hypertension, care gap, end-stage renal disease (esrd), free health clinics, chronic kidney disease (ckd)

## Abstract

Chronic kidney disease (CKD) is a condition that involves the deterioration of renal function over the course of months to years. Various clinical manifestations occur at the initial insult to the kidney, ranging from subtle changes in metabolic and volume control to asymptomatic hematuria, hypertension, and diabetes. The kidneys can adapt to damage or injury, but if left untreated, then there is a possibility of a gradual decline in renal function that progresses to kidney failure that requires dialysis. The rate of progression between stages of CKD is based upon the underlying disease, presence of comorbidity conditions, treatments, socioeconomic status, genetics, and ethnicity. If an individual’s renal function progresses to kidney failure, then patients may experience a constellation of signs and symptoms that include hyperkalemia, volume overload, hypertension, anemia, and bone disorders. Classification or staging of CKD provides a guide to management and stratification of risk for progression to kidney failure.

In this report, we describe a 47-year-old African American male who reported a 25-year history of intermittent homelessness, cocaine, and heroin use but remained free from drug use for 10 years before presenting to our clinic. The patient was diagnosed with hypertension and stage 3 kidney disease in his 30s but was unable to have regular follow-up appointments with a physician due to a lack of access to care. The patient presented asymptomatic with an estimated glomerular filtration rate of 14 mL/min and creatinine of 5.42 mg/dL. We stabilized his hypertension and consulted nephrology to assess the need and timing for dialysis. Once approved for Medicare, the patient was able to be seen within 72 hours and started on dialysis shortly after. He is currently awaiting a kidney transplant.

In this case, we describe and highlight the gaps in care for the medically uninsured, specifically patients with CKD. Our patient was diagnosed with stage 3 kidney disease 17 years before presenting to the Gary Burnstein Clinic. The gaps in accessible healthcare prevented him from accessing treatments he desperately needed. We also highlight the achievements and barriers free health clinics face on a day-to-day basis when trying to manage complex medical needs. We were able to provide high-quality healthcare to bridge the gap in access to care and ultimately get the patient the proper treatment.

## Introduction

Chronic kidney disease (CKD) is defined by the presence of kidney damage or decreased kidney function for three or more months, irrespective of the cause. The Kidney Disease Improving Global Outcomes (KDIGO) CKD guidelines diagnose CKD as decreased estimated glomerular filtration rate (eGFR) (<60 mL/min/1.73 m^2^) and at least one marker of kidney damage (albuminuria, structural abnormalities, urine sediment abnormalities, electrolyte abnormalities, histological abnormalities, and previous history of kidney transplant) for at least three months [1]. Hypertension and diabetes are the most common causes of CKD [2]. However, CKD can also be caused by glomerulonephritis [2]. To determine the cause of CKD, the initial workup should include a full medical history, physical examination, blood pressure history, dietary history, weight measurements, serum electrolytes, fasting lipids, glycated hemoglobin (HbA1c), and urine albumin/creatinine ratio [3]. Smoking, hypertension, and obesity are risk factors for developing CKD [1].

CKD staging 1-5 is determined by GFR and degree of albuminuria. According to clinical guidelines, CKD progresses to kidney failure when GFR is less than 15 mL/min/1.73 m^2^ or when a patient needs transplantation or dialysis [4]. Kidney failure does not imply end-stage renal disease (ESRD). The term ESRD is used by health insurance to define when a patient is on dialysis or transplantation and does not include patients with kidney failure not on dialysis or transplantation [4].

## Case presentation

A 47-year-old African American male presented to the Gary Burnstein Clinic to establish care. The patient reported a 25-year history of on and off homelessness, cocaine and heroin use for years but clean for the last 10 years and regularly attends narcotics anonymous. The patient reported being diagnosed with hypertension and stage 3 kidney disease when it was found he had proteinuria in his 30s but was unable to follow up with a physician regularly until he established care at our clinic.

The patient was obese with a BMI of 49.1 kg/m^2^ and complained of fatigue, daytime sleepiness, intermittent dyspnea, and pain in his toes. He denied any oliguria, nausea, vomiting, pruritis, or dysgeusia. His physical examination was consistent with uncontrolled essential hypertension with a systolic blood pressure of 186 mmHg and diastolic blood pressure of 109 mmHg. He had slight 2+ pitting edema to both extremities, but the remainder of the examination was normal. His metabolic panel was consistent with kidney failure with a GFR of 14 mL/min, blood urea nitrogen 42 mg/dL, creatinine 5.42 mg/dL, and albumin of 1563 mg/g. The lipid panel showed low levels of high-density lipoprotein cholesterol of 24 mg/dL and high levels of triglycerides of 250 mg/dL (Table 1). The patient also had a positive hepatitis C antibody and hepatitis B core antibody but negative viral titers and negative hepatitis B surface antigen, respectively (Tables 2, 3). We were able to obtain some records from physician encounters years before the patient became a patient at our clinic. We saw that the patient had a consistent eGFR value of 14 mL/min for a couple of years before our first encounter.

**Table 1:** Lipid panel HDL-C: high-density lipoprotein cholesterol; LDL-C: low-density lipoprotein cholesterol.

Lab component	Value	Reference
Cholesterol	105 mg/dL	<200
HDL-C	24 mg/dL	>40
Triglycerides	250 mg/dL	40-150
LDL-C	31 mg/dL	0-99

**Table 2:** Hepatitis B panel HBV: hepatitis B virus

Lab component	Value	Reference
Hepatitis B Surface Antibody Qualitative	Positive	Unvaccinated: Negative; Vaccinated: Positive
Hepatitis B Surface Antibody Quantitative	71.88	Unvaccinated: <12 mIU/mL; Vaccinated: >12 mIU/mL
Hepatitis B Surface Antigen	Negative	Negative
Hepatitis B Core Antibody, Total	Positive	Negative
Hepatitis B Core Antibody, IgM	Negative	Negative
Hepatitis B Virus DNA	Not Detected	Not Detected
Hepatitis B Virus DNA, Quantitative	<10 IU/mL	<10
Log HBV	<1.00 Log IU	<1.00

**Table 3:** Hepatitis C panel HCV: hepatitis C virus; EIA: enzyme immunoassay.

Lab component	Value	Reference
Hepatitis C Antibody (Third-Generation EIA)	Positive	Negative
Hepatitis C Virus RNA	Not Detected	Not Detected
Hepatitis C Virus RNA, Quantitative	<12 IU/mL	<12
Log HCV	<1.08 Log IU	<1.08

We started the patient on amlodipine 10 mg, losartan 100 mg, spironolactone 25 mg, and carvedilol 25 mg for hypertension and ESRD management as well as atorvastatin 20 mg for dyslipidemia. The patient was compliant with all his prescriptions and treatment plans. Due to his eGFR level of 14 mL/min, we kept close follow to monitor his renal disease. The patient’s eGFR classified him with ESRD and given the likelihood that his renal functions would decline further and require dialysis we had the patient apply for Medicare through the state. The patient was denied insurance multiple times and the reason given was that the patient's eGFR was not at a critical level requiring dialysis which directly contradicted the guidelines for kidney failure patients to receive Medicare.

During this time period, while trying to obtain Medicare, we continued to monitor the patient’s progression. During a follow-up blood work, we found that the patient’s renal function had progressed from an initial creatinine of 5.42 mg/dL and eGFR of 14 mL/min to creatinine of 6.87 mg/dL and eGFR of 11 mL/min between April and August. Then it progressed further to creatinine of 9.74 mg/dL and eGFR 6 mL/min from August to November (Table 4).

**Table 4:** Trending renal panel BUN: blood urea nitrogen; GFR: glomerular filtration rate.

Component	April	August	November	January	Reference range
Sodium	139 mEq/L	138 mEq/L	138 mEq/L	135 mEq/L	135-144
Potassium	5.3 mEq/L	4.8 mEq/L	4.8 mEq/L	3.8 mEq/L	3.5-5.3
Calcium	9.3 mg/dL	9.3 mg/dL	9 mg/dL	9.8 mg/dL	8.5-10.5
Phosphorus	3.7 mg/dL	4.5 mg/dL	4.6 mg/dL	5.5 mg/dL	2.5-4.6
BUN	42 mg/dL	56 mg/dL	38 mg/dL	50 mg/dL	6-23
Creatinine	5.42 mg/dL	6.87 mg/dL	9.74 mg/dL	12.07 mg/dL	0.40-1.40
GFR-Black	14 mL/min	11 mL/min	7 mL/min	5 mL/min	>60
GFR-Other	11 mL/min	9 mL/min	6 mL/min	4 mL/min	>60

The patient’s renal functions declined drastically between April and November. We began having difficulty managing his blood pressure and the patient complained of worsening peripheral edema, shortness of breath, and oliguria. At this point the patient became uremic and we had no other option but to have him go to a nearby emergency department in hopes of receiving dialysis but due to the cost of an ER visit and the inevitable hospital admission, the patient declined and opted to wait until he was approved for Medicare. Before being approved for Medicare, we had already established follow-up appointments with a nephrologist at a nearby hospital system, but care would be out of pocket. Unfortunately, the patient was unable to afford the cost of nephrologist care before his Medicare benefits were active.

Ultimately the patient was approved for Medicare insurance when his GFR dropped to 6 mL/min and was seen within 72 hours by the nephrology referral we had established. He started on hemodialysis as the mode of renal replacement therapy via tunneled catheter while arranging for arteriovenous graft and graft maturation. The patient tolerated dialysis well and has never missed an appointment. The cause of the patient’s kidney failure was likely due to hypertension and clinically suspicious for focal segmental glomerulosclerosis, which is most likely due to his obesity, although no biopsy was performed for confirmation. The patient was classified with the Karnofsky tool that classifies patients to their functional impairment and was placed at 50%.

Due to Medicare gaps in coverage, the patient was still coming to our clinic for management of his hypertension and hypercholesteremia. It was difficult to maintain open and accurate lines of communication between his specialist physician teams treating his kidney failure and ours to ensure that there was no contradicting or conflicting information potentially leading to costly errors and/or polypharmacy.

The patient is currently married and has two children. He has strong social support, and the patient has an unyielding desire to comply with treatment. At the time of this paper, the patient is actively waiting for transplantation. He continues to receive dialysis every Monday, Wednesday, and Friday and has not missed an appointment. He remains serious and committed to his sobriety and has a positive outlook on the future and his life.

## Discussion

CKD can bear a significant financial burden and varies based on staging. For patients with CKD stages 4 and 5, Medicare and private insurer spending can vary from $7,000 to $65,000 per patient [5]. The majority of costs can be attributed to inpatient care (acute events, preparing for kidney transplantation, or dialysis) [5]. If CKD progresses to ESRD, the costs are $65,312 per patient on average for Medicare and $96,000 to $180,000 per patient for private insurers [5]. Despite making up less than 1% of the Medicare population, care of patients with CKD costs roughly 7% of the Medicare budget [6]. The high costs of ESRD are attributed to dialysis preparation, dialysis therapy, and complications with reduced renal function [5].

While Medicare covers patients with advanced CKD requiring dialysis, there is no health coverage for patients with earlier stages of CKD that are younger than 65 and do not have a disability qualifying them for Medicare [6]. Consequently, this leads many patients to delay seeing a nephrologist. Uninsured CKD patients are significantly more likely to be referred late to nephrologists than patients with insurance [7]. Patients unhoused or unemployed are also significantly more likely to be referred later [7]. According to clinical guidelines, a patient should be under the care of a nephrologist when their GFR drops below 30 mL/min/1.73 m^2^ (stage 3). However, uninsured patients’ ineligibility for Medicare often cannot and will not follow this recommendation due to cost, which can cause their CKD to progress to a more advanced disease [4].

The non-white race is a widely recognized risk factor for the progression of CKD to ESRD [6]. Black CKD patients have a four times greater risk of progressing to ESRD, Asians have a two times greater risk, and Hispanics have a 1.5 times greater risk than non-Hispanic whites [6]. One contributing factor is the race-based disparity in health insurance. According to the United States, Renal Data System, 11.4% of all Blacks, 39.7% of all Hispanics, and 8.2% of all Asians and Pacific Islanders lack health insurance at the beginning of their ESRD, in contrast to 5.8% of whites [6]. Even though CKD is generally more common in older persons, CKD is more likely to progress to ESRD in younger persons [6]. Possible causes include the risk of death in older persons before progression to ESRD, as well as a potentially faster decline in renal function in younger persons [6]. Uninsured persons with CKD are on average 18 years younger than insured persons with CKD [6]. Therefore, uninsured patients are more likely to progress to ESRD. 

Delaying care with a nephrologist is associated with a greater risk of unplanned first dialysis, more complications, greater hospital costs, and increased length of hospitalization within the first three months of dialysis [8]. Additionally, patients who are uninsured are not only less likely to see a nephrologist, but less likely to have access to a primary care physician that can manage early complications of their CKD. Hypertension can cause CKD and is also a complication of CKD that needs to be controlled in all patients, regardless of CKD stage [4]. Other manifestations of CKD that typically start in stage 3 such as anemia, malnutrition, bone disease, and neuropathy must be monitored and treated. Kidney replacement therapy preparation is recommended to start during stage 4 [4]. Risks of death, cardiovascular events, and hospitalization have been found to increase with declines in GFR [9].

## Conclusions

This case highlights the gaps in care for uninsured patients with CKD. While our patient was diagnosed with hypertension and stage 3 kidney disease in his 30s, his health coverage status prevented him from regularly accessing care for his hypertension and seeing a specialist at an earlier stage of kidney disease. Additionally, despite having kidney failure for years (GFR of 14 mL/min), Medicare was not approved until his GFR deteriorated to 6 mL/min. For uninsured patients, starting Medicare coverage until they are considered ESRD causes many patients to forego much-needed care in the earlier stages of CKD. By treating patients prior to kidney failure, Medicare can potentially avoid the high costs of ESRD and improve morbidity and mortality for many uninsured CKD patients.
